# Future Intentions of Fitness Center Customers: Effect of Emotions, Perceived Well-Being and Management Variables

**DOI:** 10.3389/fpsyg.2020.547846

**Published:** 2020-09-25

**Authors:** Fernando García-Pascual, Vicente Prado-Gascó, Mario Alguacil, Irena Valantine, Ferran Calabuig-Moreno

**Affiliations:** ^1^Department of Physical Education and Sports, University of Valencia, Valencia, Spain; ^2^Department of Social Psychology, University of Valencia, Valencia, Spain; ^3^Department of Teaching and Learning of Physical, Plastic and Musical Education, Catholic University of Valencia, Valencia, Spain; ^4^Department of Sport Management, Economics and Sociology, Lithuanian Sports University, Kaunas, Lithuania

**Keywords:** future intentions, sports center, emotions, well-being, QCA, satisfaction, perceived value, sport management

## Abstract

One of the main objectives of fitness center managers is to obtain high levels of loyalty from the customers of these fitness centers. Within the existing literature on fitness center management, previous research has analyzed the importance of the management variables themselves to determine the behavioral intentions of their customers, ignoring other psychological and sociodemographic aspects and focusing on linear relationship models. Therefore, this study, which aims to analyze the impact of different management variables along with psychological (emotions and subjective well-being) and demographic variables (age and sex) on the satisfaction, perceived value (PV) and future intentions (FI) of 398 users (216 men, aged 18–75, Mean = 35.89 ± 14.53) of a fitness center, intends to fill this gap. In this study, two different methodologies are used, i.e., hierarchical regressions models (HRM) and qualitative comparative analysis (QCA). The data were obtained by means of a self-administered questionnaire composed of 69 items that collected different previously validated scales. Considering HRM, the different models proposed explain 52% of the satisfaction variance, 57% of perceived value and 59% of future intentions. In general, it seems that the management variables are better predictors than subjective well-being, emotions and age or gender since their inclusion does not greatly improve the model’s predictive capacity. As far as QCA analyses are concerned, it seems that none of the conditions are necessary for high or low levels of satisfaction, perceived value or future intentions. On the other hand, based on sufficiency analyses, there seem to be 8 pathways or combinations of conditions leading to high levels of satisfaction and 13 for low levels, 11 combinations leading to high levels of IF and 10 leading to low levels; however, there are 6 combinations of conditions leading to high levels of PV and 5 leading to low levels. In general, some of the pathways consider only the management variables, although many of them consider the importance not only of the management variables but also of the interactions that these may have with emotional aspects and, to a lesser extent, with age and well-being. When comparing both methodologies, it can be observed that the QCA models are more explanatory than the HRM models and that they take into account aspects that seem unimportant when observing linear models (such as emotions or age). However, both methodologies should be considered complementary and used simultaneously since, by focusing on different aspects, enriched results are obtained. The results obtained will enable managers to make more efficient use of available resources to increase user satisfaction.

## Introduction

In recent times, sports or fitness centers have become predominantly important in the field of sports services. In Spain, the increase in these services has contributed to the country being placed in fifth position within the European context in terms of economic income. Such is the importance, that in 2018, it generated 2,291 million euros, a 2.5% increase over the previous year (Deloitte, 2019). On the one hand, these data reveal the importance of this sector for the economy, but on the other hand, these centers have a very important role in the health of those who use them. Understanding health not only in regard to physiological effects on the body but also psychological aspects, such as subjective well-being or positive emotions caused by the practice of sports, is important.

The increase in the turnover in the fitness sector implies the improvement of the system; the number of competitive sport centers with optimal market shares is increasing, as this quality/price relationship is one of the most observed factors influencing sport center users (Gallardo et al., 2016). In these centers, the customers’ needs regarding not only sports but also their social and psychological are satisfied; consequently, the loyalty of the customer is prolonged, which is the aim of all sports services. In their study, Molina et al. (2016) affirm that the emotional value is the variable that most influences the future intentions of fitness centers customers.

The practice of sports is one of the main elements in the physical and psychological well-being of society (Teixeira et al., 2012). According to the latest available survey on sports habits in Spain (2015), the health motive is the third main reason for practicing sport, with women scoring highest on this motive. On the other hand, this same survey explains that more than half of the Spanish society over 15 years of age participates in physical activities at least once every 3 months. At a European level, Eurostat, places Spain above the European Union average in the practice of sport, with 34% of society doing sport more than 2.5 h a week, even ahead of countries such as France and Italy. Therefore, fitness centers are important in satisfying both the physical and psychological needs of sports customers. These sports centers, whose priority objective is customer loyalty, try to strengthen this bond through different service adaptation mechanisms.

In their paper, Gallardo et al. (2016) argue that lack of time, value for money and motivation are the main causes of the drop in attendance at sports centers. Therefore, it is important to adapt these sports services to the demands of the users to further strengthen their feelings of loyalty. Determining which variables predict loyalty or help loyalty to appear while also helping to strengthen it is a very important task that these sports centers must perform. Within the context of these centers, there are studies (De Knop et al., 2004; Calabuig et al., 2012b; García-Fernández et al., 2012) that have analyzed management variables such as service quality and customer satisfaction. The quality of service is a strong antecedent of customer satisfaction, so these sports services increasingly offer renewed quality systems (García-Fernández et al., 2018a). Thus, they are adjusted to the profiles of the clients who attend the sports center to reinforce the satisfaction that the clients feel with the service. Customer satisfaction has been one of the most frequently analyzed variables in sports management (Alcañiz and Simó, 2004; Yoshida and James, 2010; Baena-Arroyo et al., 2016; Dias et al., 2019) and the significance of this variable is such that the service customer’s future behavioral intentions may be highly influenced by unsatisfactory experiences (Morales and Hernández, 2004).

These management variables, as well as the perceived value of the service, have been used to try to measure the customer’s perceptions in relation to their future behavior, that is, their intentions to return to these sports centers (García-Fernández et al., 2018a). Therefore, for the sports managers who run these sports facilities, customer loyalty is one of the greatest challenges they face (García-Pascual et al., 2020). Different studies confirm the close relationship between management variables and the future behavioral intentions of sports center customers (García-Fernández et al., 2016; Chiu et al., 2019; Baena-Arroyo et al., 2020).

Proposition 1. Satisfaction is a predictor of future customer intentions.

Within sports organizations, Calabuig et al. (2008), argue that customer satisfaction is one of the most analyzed management variables because it offers a result in which the customer, in a subjective way, measures his experience with that organization. Within the existing literature, in the field of sports facilities, there are different studies that confirm the recognized link between customer satisfaction and the future behavioral intentions of the customer (Calabuig et al., 2012a; García-Fernández et al., 2018b; Alguacil et al., 2019). Likewise, Avourdiadou and Theodorakis (2014), in their research, analyze the perceptions of sports center customers in Greece, concluding that customer satisfaction is the main driver of these customers’ future behavioral intentions.

Proposition 2. Perceived value is a predictor of the customer’s future intentions.

The perceived value of the customer is a variable that is very much analyzed in the literature of sports services (Molina et al., 2016; Chiu et al., 2019), as well as being a strong antecedent of customer satisfaction. With regard to sports centers, the literature includes different studies that argue that this variable, the perceived value of the customer, is a clear antecedent of the behavioral intentions of customers (Brady et al., 2005; Lu et al., 2011). Likewise, Yu et al. (2014), in their research, analyze this link between these variables, stating that the greater the perception of value, the greater loyalty the service will achieve.

Proposition 3. Emotions influence the predicted future behavioral intentions of customers.

Emotions in sport management have mostly been analyzed within the context of sport events, i.e., analyzing the emotional state of the spectators when attending such event (Biscaia et al., 2012; Calabuig et al., 2015; Song et al., 2019).

On the other hand, few studies have been found that focus on sports centers, although there have been recent studies (Biscaia et al., 2012; Molina et al., 2016) analyzing psychological variables, such as life satisfaction, happiness, subjective well-being, and emotions, and their influence on customers’ perceptions of a service. Pedragosa et al. (2015) argue that both positive and negative emotions influence customer satisfaction and, consequently, influence future behavioral intentions. Similarly, Ong and Yap (2017) analyze the perceptions of customers of fitness centers in Malaysia, stating that the emotional response seems to have a stronger positive relationship with the behavioral intentions of customers.

Proposition 4. Subjective well-being is predictive of future customer intentions.

It is true that, within the literature on sport management, specifically that which studies the perceptions of customers of fitness centers, there are very few studies that deal in any way with the well-being of customers. In recent years, some scholars have analyzed this psychological variable within sports centers (Hamer and Stamatakis, 2010; García-Pascual et al., 2016; Molina, 2016). These studies affirm the close and important relationship between sports practice and personal well-being and that this psychological variable predicts the customers’ future behavioral intentions regarding the sports service.

Proposition 5. Age and gender influence the prediction of future customer intentions.

On the other hand, several sociodemographic variables have been analyzed in the literature on sport management, such as the ages and genders of customers (González-Cutre and Sicilia, 2012; Borges-Silva et al., 2017; Zamorano-Solís and García-Fernández, 2018). In this sense, García-Fernández et al. (2013) find that it is women and older customers who score highest on the dimensions of quality and loyalty to the service.

Another key aspect in the development of sports services is the segmentation of the users. In the literature, for some time now and due to the increase in the world of fitness, there have been different studies analyzing this segmentation (Elasri Ejjaberi et al., 2016; Campos et al., 2017; Molina et al., 2019; León-Quismondo et al., 2020). These studies aim to establish segmented criteria for each user profile, such as prices and different services, which give the sports center a differentiating added value within the fitness market.

The aim of this paper is to analyze the perceptions of the customers of a private sports center to determine which management variables and which psychological variables (personal well-being and emotions) influence the future intentions of customers, while also incorporating age and gender into the equation. Furthermore, these relationships will be approached from two different methodologies, with one being symmetric (hierarchical regression) and the other being asymmetric (fussy set qualitative comparative analysis).

## Materials and Methods

### Participants

The sample was made up of the customers of a private sports center belonging to a town in the province of Valencia, Spain. A non-probabilistic convenience sample was used. The data were collected in person from the customers of the sports facility between October 2018 and April 2019 to obtain the perceptions of the different customers of the same center. Finally, 411 questionnaires were collected, of which 13 were incomplete, resulting in 398 questionnaires completed correctly by customers, including 216 men and 182 women. The average age of the customers surveyed was 35.89 years (±14.53). A large proportion of those surveyed had secondary or university educations (84.9%), and almost half of those surveyed (43.7%) attend the sports facility four or more times a week.

### Questionnaire

The questionnaire used in the present research is composed of six scales that have been widely used in the literature, with a total of 69 items using a five-point Likert scale from (1) “Strongly Disagree” to (5) “Strongly Agree.” The completion time is 30 min.

The scale measuring the perceived service quality was made up of 36 indicators taken from Ko and Pastore (2005), which was adapted into the Spanish context by Molina (2016). The previous studies confirmed that this scale had good psychometric properties (i.e., Molina, 2016).

Customer satisfaction was measured by two indicators taken from Hightower et al. (2002). The previous studies also confirmed the adequate psychometric properties of the scale (i.e., García-Pascual et al., 2019).

The perceived value by customers was measured through seven indicators (Sweeney and Soutar, 2001). The psychometric properties offered by the scale have been proved (i.e., Pastor-Barceló et al., 2016).

Future intentions were measured by a scale composed of four items from Zeithaml et al. (1996). The previous studies focusing on sports centers confirm the adequate psychometric properties of the scale (e.g., Molina et al., 2016; García-Fernández et al., 2018b).

The scale that measures subjective well-being is made up of eight indicators (Cummins, 2006). The studies in the literature of sports management confirm the psychometric properties of the scale (i.e., García-Pascual et al., 2016).

To measure customers’ emotions, a scale composed of 12 items was used (Bigné and Andreu, 2004). The previous studies in the sports context confirm the adequate psychometric properties of this scale (e.g., Molina et al., 2016).

### Data Analysis

First, a descriptive analysis of the sample was performed. Second, two different methodologies were used to analyze the influence of the managerial variables, subjective well-being, emotions, and sociodemographic variables (age and sex) on consumers’ satisfaction using three hierarchical regression models (HRM) and six fuzzy-set qualitative comparative analysis models (fsQCA). The descriptive and regression analyses were performed used SPSS (Statistical Package for the Social Sciences, Version 22, IBM), and the fsQCA 2.0 software package (Claude and Christopher, 2014) was used to calculate the fsQCA models.

In the case of HRM, three models (prediction of satisfaction, perceived valued and future intentions) with four steps were performed. In the first step, the managerial variables were included, then, in the second step, subjective well-being was included; in the following step, the emotional variables (pleasure and arousal) were included, and in the fourth step, the demographic variables were also included (age and sex).

Regarding fsQCA, as suggested in the literature (Ragin, 2008), the raw data obtained were transformed into fuzzy set responses. First, the data with missing responses were deleted, then different constructs were obtained by multiplying the items that make them up (Giménez-Espert and Prado-Gascó, 2018). Third, the three thresholds of calibration were calculated according to Woodside’s (2013) suggestion, as follows: 10% (totally out of the set), 50% (neither in nor out of the set) and 90% (totally in the set). Finally, the values of all the constructs were recalibrated between 0 and 1 (Ragin, 2008) by the automated process of the fsQCA 2.5 software (Claude and Christopher, 2014). Once the data were transformed for analysis into QCA, the necessity and sufficiency analyses were carried out to predict the high levels (presence) or low levels (absence) of satisfaction, perceived valued and future intentions.

In QCA, the explained variance of the model is reflected in the coverage of the solution, while a measure of adjustment of the model it is represented through consistency (Prado-Gascó and Calabuig, 2016). A condition is considered necessary in QCA models when its consistency is greater than or equal to 0.90 (Ragin, 2008). Sufficiency analyses allow for three types of solutions, i.e., complex, parsimonious and intermediate (Eng and Woodside, 2012). The intermediate solution is the one recommended in the literature (Ragin, 2008), and it is shown here.

## Results

### Hierarchical Regression Model

Based on the HRM results, the prediction models predict between 52% and 59% of the variables under study (satisfaction: *R*^2^ = 0.52; perceived value: *R*^2^ = 0.57; future intentions: *R*^2^ = 0.59). In all cases, the management variables substantially improve the predictive capacity of the different models, while the inclusion of psychological or demographic variables improves the models very little.

As seen in Table 1, the sports management variables (SQ, SAT, PV, IF) psychological variables (subjective well-being, pleasure and arousal) and the sociodemographic variables (age and sex) explain 59% (adjusted *R*^2^ = 0.59) of the consumers’ future intentions. Analyzing the different steps that were carried out in the hierarchical regression more specifically, it can be seen how in the prediction of the future intentions, the inclusion of the sport management variables increases the predictive capacity of the model by a 58% (Δ*R*^2^ = 0.58, *p* < 0.001), while the addition of the subjective well-being in the second step, the emotions (pleasure and arousal) in the third step and the age and sex in the last step scarcely improve the model’s prediction (Δ*R*^2^ = 0.01, *p* < 0.001 in all cases). Considering the results of the last step, the variables that resulted in significant predictors were SAT (β = 0.22; *p* < 0.001), PV (β = 0.52; *p* < 0.001), SWB (β = 0.12; *p* < 0.001) and, to a much lower extent, pleasure (β = 0.09; *p* < 0.05).

**TABLE 1:** Hierarchical regression models of management variables, customer emotions, subjective well-being, age and sex in satisfaction, perceived value, and future intentions.

	Satisfaction		Perceived value		Future intentions	
			
	Δ*R*^2^	β	Δ*R*^2^	β	Δ*R*^2^	β
**Step 1**	0.51***		0.56***		0.58***	
Service quality		0.58***		0.75***		–
Satisfaction		–		–		0.25***
Perceived value		0.17***		–		0.59***
**Step 2**	0.01		0.01*		0.01**	
Service quality		0.58***		0.72***		–
Satisfaction		–		–		0.24***
Perceived value		0.17***		–		0.55***
Subjective well-being		−0.03		0.08***		0.13***
**Step 3**	0.01		0.01*		0.01*	
Service quality		0.58***		0.69***		–
Satisfaction		–		–		0.23***
Perceived value		0.17***		–		0.53***
Subjective well-being		−0.02		0.07		0.12***
Pleasure		0.06		0.10**		0.10*
Arousal		−0.09*		0.01		−0.02
**Paso 4**	0.002		0.01		0.01	
Service quality		0.58***		0.68***		–
Satisfaction		–		–		0.22***
Perceived value		0.18***		–		0.52***
Subjective well-being		−0.02		0.07*		0.12***
Pleasure		0.05		0.11**		0.09*
Arousal		0.08*		−0.01		−0.01
Age		−0.02		0.05		0.05
Man		−0.04		0.05		−0.05
**Total *R*^2^_*adj*_**	0.52***		0.57***		0.59***	

Δ*R*^2^, *R*-square change; β, standardized beta; *R*^2^_*adj*_, *R*-square adjusted; *p ≤ 0.05; **p ≤ 0.01; ***p ≤ 0.001.

The ways that the management variables (SQ, VP), psychological variables (subjective well-being, pleasure and arousal) and sociodemographic variables (age and sex) explain 52% (adjusted *R*^2^ = 0.52) of user satisfaction were also observed. In the first step, in predicting the level of user satisfaction, the management variables increase the model’s prediction by 51% (Δ*R*^2^ = 0.52, *p* < 0.001), observing that in the successive steps, when emotions (pleasure and arousal), subjective well-being and sociodemographic variables are incorporated, the model’s prediction increases very slightly (Δ*R*^2^ = 0.01, *p* < 0.001 in all cases). Observing the last step, the variables that showed significance in predicting the satisfaction of the users of the sports center were SQ (β = 0.58; *p* < 0.001), PV (β = 0.18; *p* < 0.001) and arousal (β = 0.08; *p* < 0.05).

Finally, in the prediction of the perceived value of the users, the management variables (SQ) the psychological variables (subjective well-being, pleasure and arousal) and the sociodemographic variables (age and sex) explain 57% (adjusted *R*^2^ = 0.57) of the predicted value. Analyzing the different steps of this hierarchical regression, it is observed that in the first step, the sports management variable increases the predictive capacity of the model by 56% (Δ*R*^2^ = 0.56, *p* < 0.001), and in the following steps, when emotions (pleasure and arousal), subjective well-being and sociodemographic variables are incorporated, the model’s prediction increases (Δ*R*^2^ = 0.01, *p* < 0.001 in all cases). In the last step, the variables that showed significance in the prediction of the users’ perceived value are observed, i.e., SQ (β = 0.68; *p* < 0.001), pleasure (β = 0.11; *p* < 0.01) and subjective well-being (β = 0.07; *p* < 0.05).

It is observed that the management variables are those that predict in a differentiated way the dependent variables of this research, with a very slight improvement of the model occurring with the incorporation of the subjective well-being, emotions or the two sociodemographic variables.

### Qualitative Comparative Analysis (fsQCA)

First, the descriptive statistics and the three thresholds for the calibration values of the variables are calculated and then analyzed (Table 2).

**TABLE 2:** Descriptive statistics and calibration values.

		Age	SQ	SAT	PV	FI	SWB	Pleasure	Arousal
Mean		35,89	361940,89	17,54	18774,54	327,80	22390606,11	5980,56	3110,27
SD		14,53	362997,07	5,70	20976,66	197,81	28275760,86	4894,99	3649,48
Minimum		18	1	1	1	8	4500	36	9
Maximum		77	1562500	25	78125	625	100000000	15625	15625
Percentiles	10	20	46210,2	9	1728	81	838635	1116	404
	50	32	245367,5	16	11520	256	10266480	4096	1728
	90	58	886936,25	25	50000	625	66249000	15625	7500

*SD, standard deviation; SQ, service quality; SAT, satisfaction; PV, perceived value; FI, future intentions; SWB, subjective well-being.*

Then, the necessity analysis was carried out to determine if there were causal conditions for the presence or absence (∼) of the analyzed variables that were individually necessary. As shown in Table 3, none of the conditions are necessary since the consistency of each one of them does not present results above 0.90 (Ragin, 2008).

**TABLE 3:** Necessity analysis for satisfaction, perceived value, and future intentions.

	SAT		∼SAT		PV		∼PV		FI		∼FI	
	Cons	Cov	Cons	Cov	Cons	Cov	Cons	Cov	Cons	Cov	Cons	Cov
SQ	0.71	0.87	0.43	0.41	0.78	0.76	0.43	0.52	–	–	–	–
∼SQ	0.52	0.54	0.86	0.70	0.51	0.42	0.80	0.82	–	–	–	–
SAT	–	–	–	–	–	–	–	–	0.82	0.76	0.54	0.46
∼SAT	–	–	–	–	–	–	–	–	0.42	0.51	0.72	0.78
PV	0.68	0.85	0.44	0.43	–	–	–	–	0.73	0.86	0.39	0.41
∼PV	0.55	0.56	0.85	0.67	–	–	–	–	0.50	0.47	0.87	0.74
SWB	0.59	0.74	0.52	0.51	0.67	0.66	0.47	0.58	0.62	0.73	0.48	0.51
∼SWB	0.61	0.62	0.74	0.58	0.58	0.47	0.73	0.73	0.58	0.55	0.75	0.64
Pleasure	0.64	0.76	0.54	0.50	0.70	0.66	0.51	0.60	0.67	0.74	0.50	0.51
∼Pleasure	0.58	0.62	0.74	0.61	0.57	0.48	0.7	0.74	0.55	0.55	0.74	0.67
Arousal	0.59	0.70	0.57	0.54	0.63	0.60	0.54	0.64	0.60	0.67	0.55	0.56
∼Arousal	0.61	0.65	0.68	0.56	0.62	0.52	0.66	0.69	0.61	0.60	0.68	0.61
Age	0.59	0.70	0.57	0.52	0.63	0.59	0.55	0.64	0.61	0.67	0.54	0.54
∼Age	0.59	0.64	0.67	0.56	0.61	0.52	0.64	0.68	0.58	0.58	0.67	0.61
Men	0.51	0.53	0.57	0.46	0.53	0.44	0.54	0.55	0.51	0.49	0.57	0.50
∼Men	0.48	0.59	0.42	0.40	0.46	0.45	0.45	0.54	0.48	0.55	0.42	0.44

*∼, absence of condition; Con, consistency; Cov, coverage; SQ, service quality; SAT, satisfaction; PV, perceived value; FI, future intentions; SWB, subjective well-being. Condition needed: consistency ≥ 0.90. –, do not calculated according to the theoretical model.*

Finally, the sufficiency analysis was carried out to determine if the conditions that make up the model are sufficient. Table 4 presents the three most important combinations of condition for the intermediate solution. Analyzing Table 4, it can be seen that the consistencies of the different combinations were adequate, as all are above the minimum recommended cut-off point of 0.75 (Ragin, 2008).

**TABLE 4:** Three main conditions of sufficiency analysis for satisfaction, perceived value, and future intentions (intermediate solution).

*Frequency cutoff: 1*			SAT			∼SAT			PV			∼PV			FI			∼FI		

			*Consistency cutoff: 0.85*			*Consistency cutoff: 0.85*			*Consistency cutoff: 0.83*			*Consistency cutoff: 0.85*			*Consistency cutoff: 0.86*			*Consistency cutoff: 0.85*		

			1	2	3	1	2	3	1	2	3	1	2	3	1	2	3	1	2	3
																				
Service quality			•			◯	◯	◯	•	•	•	◯			–	–	–	–	–	–
Satisfaction			–	–	–	–	–	–	–	–	–	–	–	–	•		•	•		
Perceived value				•		◯	◯	◯	–	–	–	–	–	–	•	•		•	•	•
Future intentions			–	–	–	–	–	–	–	–	–	–	–	–	–	–	–	–	–	–
Subjective well-being							•	•	•					◯			•		◯	
Pleasure					•			◯		•			◯	◯				◯	◯	◯
Arousal					•		•			•	•			◯			◯			
Men						•										•				•
Age					•			◯		◯	•		◯						◯	
Raw coverage			0.71	0.68	0.32	0.45	0.31	0.30	0.58	0.32	0.30	0.81	0.49	0.36	0.66	0.39	0.36	0.55	0.42	0.41
Unique coverage			0.08	0.07	0.01	0.12	0.01	0.02	0.11	0.01	0.01	0.25	0.03	0.01	0.01	0.02	0.02	0.01	0.03	0.01
Consistency			0.88	0.86	0.80	0.77	0.86	0.87	0.84	0.85	0.84	0.82	0.82	0.90	0.90	0.85	0.87	0.89	0.89	0.83
**Overall solution consistency**					**0.79**			**0.75**			**0.81**			**0.77**			**0.80**			**0.81**
**Overall solution coverage**					**0.86**			**0.72**			**0.72**			**0.88**			**0.84**			**0.79**
																				

*∼, absence (low levels) of condition; •, presence (high levels) of condition; ◯, absence (low levels) of condition. SAT, satisfaction; PV, perceived value; FI, future Intentions. –, do not calculated according to the theoretical model. Expected vector for satisfaction: 1.-0.1.-0.1.1.1.1.0 (0: absent; 1: present), expected vector for ∼ satisfaction: 0.-0.0.-0.0.0.0.0.1 (0: absent; 1: present), expected vector for perceived value: 1.-.-.-0.1.1.1.1.0 (0: absent; 1: present), expected vector for ∼ perceived value: 0.-.-.-0.0.0.0.0.1 (0: absent; 1: present), expected vector for future intentions: -0.1.1.1.1.1.1.1.0 (0: absent; 1: present), expected vector for ∼ future intentions: -0.0.0.0.0.0.0.0.1 (0: absent; 1: present).*

Therefore, considering the sufficiency analysis, the different combinations or paths that result explain the different models. Regarding the future intentions of the customers, 11 combinations explain 84% of FI (coverage solution: 0.84; consistency solution: 0.80); the three main important combinations for the presence of FI are SAT^∗^PV (raw coverage: 0.66; consistency: 0.90), PV^∗^Men (raw coverage: 0.39; consistency: 0.85), and SAT^∗^SWB^∗^∼arousal (raw coverage: 0.36; consistency: 0.87). There are also 10 combinations of conditions that lead (79%) to low levels of FI (solution coverage: 0.79; solution consistency: 0.81); the three most important combinations are ∼SAT^∗^∼PV^∗^∼arousal (raw coverage: 0.55; consistency: 0.89); ∼PV^∗^∼SWB^∗^∼AROU^∗^being younger (raw coverage: 0.42; consistency: 0.89); and ∼PV^∗^∼arousal^∗^Men (raw coverage: 0.41; consistency: 0.83).

To measure the customer satisfaction variable, eight pathways or combinations explain 86% of the high levels of SAT (coverage solution: 0.86; consistency solution: 0.79), with three combinations being the most important, i.e., SQ (caw coverage: 0.71; consistency: 0.88), ∼PV (coverage: 0.68; consistency: 0.86), and AROU^∗^PLEA^∗^AGE (raw coverage: 0.32; consistency: 0.80). On the other hand, there are 13 combinations of conditions (72%) indicating low levels of SAT (coverage solution: 0.72; consistency solution: 0.75); the three more important for the absence of SAT are ∼SQ^∗^∼PV^∗^MAN (raw coverage: 0.71; consistency: 0.88), ∼SQ^∗^∼PV^∗^PWB^∗^AROU (raw coverage: 0.66; consistency: 0.90), and ∼SQ^∗^∼PV^∗^PWB^∗^∼PLEA^∗^∼AGE (raw coverage: 0.66; consistency: 0.90).

Finally, the variable analyzed of the value perceived by the customer results in six combinations of conditions explaining 72% of PV (coverage solution: 0.72; consistency solution: 0.81); the three main important combinations for the presence of PV are SQ^∗^BS (raw coverage: 0.58; consistency: 0.84), SQ^∗^PLEA^∗^AROU^∗^∼AGE (raw coverage: 0.32; consistency: 0.85), and SQ^∗^AROU^∗^AGE (raw coverage: 0.30; consistency: 0.84). On the other hand, there are also five combinations of conditions that explain 88% of the low levels of PV (coverage solution: 0.88; consistency solution: 0.77); three main important combinations for the absence of PV are ∼SQ (raw coverage: 0.81; consistency: 0.82), ∼PLEA^∗^∼AGE (raw coverage: 0.49; consistency: 0.82), and ∼PWB^∗^∼PLEA^∗^∼AROU (raw coverage: 0.36; consistency: 0.90).

## Discussion

Within the context of sports centers and their management, customers’ perceptions allow information to be obtained about their opinions of the services offered. Therefore, in the literature, during the last decades, management models such as perceived quality, satisfaction or perceived value have been commonly used to predict costumers’ future intentions. However, for some time now, there have been variables in different studies that are becoming vitally important in predicting future intentions, among which, we find the emotions-related variables (Pedragosa et al., 2015; Molina et al., 2016; Ong and Yap, 2017). Likewise, these different studies have focused on analyzing, through linear models, these perceptions, without considering if in a combinatorial way there are variables that explain the prediction of an outcome variable more broadly. Some authors have attempted to solve this lack of knowledge about the interaction of variables by combining methodologies, which is why studies such as those of Calabuig et al. (2015) or Alguacil et al. (2019) analyze the sports context by combining HRM and QCA. Specifically, the article by Alguacil et al. (2019) analyses the future intentions of the users of sports services, where, as in this study, the importance of satisfaction and perceived value is confirmed, showing a direct influence on the regression models and also being part of some of the combinations that produce the expected results, as in our case, the improvement of future intentions. This influence of satisfaction and perceived value on future intentions is a connection confirmed throughout the literature (Chen and Chen, 2010). Therefore, in this research, we have used qualitative comparative analysis (fsQCA), a technique that allows one to know the combination of causal conditions that explain a specific outcome (equifinality) since this is a method of intersections where we know the contribution of each variable and the relationship between them in the prediction of a variable. In recent years, there has been a substantial increase in the number of studies using this methodology as part of the analysis of data in the field of sports management (Prado-Gascó et al., 2017; Alonso-Dos-Santos et al., 2018; Clausen et al., 2018; Väätäinen and Dickenson, 2019).

Later, in the sufficiency analysis, we have seen the role of different variables to achieve the result of interest. Although the analysis of these variables is not very common from this methodological approach, and therefore some relationships do not have references, we do observe some of the relationships proposed coincide with those stated in literature. In these analyzed combinations, we have seen, for instance, that the combination of high satisfaction and a high perceived value of the service by the customers is a background for their future intentions, this influence of satisfaction and perceived value on future intentions has been widely supported in the literature (Murray and Howat, 2002; Alexandris et al., 2006; Calabuig et al., 2015; Hyun and Jordan, 2020) so in this sense, it agrees with our study, although there are few examples in which these variables are analyzed from the methodological approach used in this research (Alguacil et al., 2019). Besides, it has also been possible to observe the relationship between well-being and future intentions (Alvarez et al., 2012; Mirehie and Gibson, 2020), in line with this study.

On the other hand, the combinations that explain low levels of future behavioral intentions with the sports service, include low levels of satisfaction and perceived value, as well as an absence of positive emotions. In this sense, in the same way that perceived quality and satisfaction predict future intentions, as discussed above, it seems logical, therefore, that low levels of these variables offer low levels of future intentions, in addition to the role of emotions, which throughout the literature have been shown to be related to future intentions (Cho and Lee, 2019; Foroughi et al., 2019; Prayag et al., 2020).

Finally, the relationship of perceived quality and perceived value to satisfaction has also been demonstrated (Shonk and Chelladurai, 2008; Nuviala et al., 2012; Howat and Assaker, 2013; García-Fernández et al., 2018a) as well as the role of perceived value in enhancing the well-being (García-Pascual et al., 2016).

The analysis of the hierarchical regression shows that the management variables have the greatest weight in predicting customers’ future intentions, with the perceived value having the greatest weight in this prediction, a result that has also been obtained in different studies (Calabuig et al., 2015). However, this the hierarchical regression result shows the individual contribution of each variable, while the interactions between variables that may occur in the prediction of management models are not known.

On the other hand, observing the fsQCA analysis, none of the conditions analyzed are necessary to predict a dependent variable; however, within the sufficiency analysis, the causal conditions within the different combinations that form the future intentions of the customers are observed, and satisfaction and perceived value are the variables present in all of them. These two variables favor high levels of future behavioral intentions with the sports service, and research within the sports management literature that argues these results has been found (Yacout, 2010; García-Fernández et al., 2018b). Analyzing the results obtained both in the linear model and in the fsQCA, although there are different studies that support the determination of fsQCA to obtain more explanatory results that show indicators, such as the constancy of the model, as far as the methodology within the field of sports management is concerned, and specifically the sports services and the variables that make it up, both methodologies should be used since they are complementary procedures (Crespo-Hervás et al., 2019).

## Conclusion

Regarding the conclusions, in the prediction of future behavioral intentions, in both the symmetric and asymmetric methodologies, satisfaction and perceived value are relevant. Even within the asymmetric analysis, it can be seen how both variables are considered as necessary in the prediction of future customer intentions. On the other hand, it is observed that the psychological variables (subjective well-being and emotions) do not play very relevant roles in predicting customers’ behavioral intentions. However, it is observed that within the combination sets analysis (fsQCA), regarding the low levels of future behavioral intentions on the part of the customers of the sport service, these psychological variables do have a participative role. It can be understood that low levels of these psychological variables (emotions and well-being) imply that there are low levels in the perception of loyalty on the part of the customers of the sports center.

These findings indicate that sports managers must propose ways to try to increase the degree of satisfaction of customers, as well as increase the perception of value, in a positive way. On the other hand, as the emotional variables are part of some combinations that achieve the expected result, sports center managers, through employees and programs, must promote activities that emphasize and reinforce positive emotions, as well as achieve positive levels of well-being.

## Managerial Implications

For sports managers, knowing the extent to which certain variables can affect or not the future intentions of users is a key aspect of ensuring the sustainability of the service. In this sense, analyzing variables, such as those related to emotional aspects, which have not been studied extensively in the literature, clarifies whether some recommendations are as relevant as they might seem. Therefore, this research provides new information for managers, with the intention of understanding the existence of determinants that have not been considered and which may be interesting for improving user behavior. Through studies such as the one carried out here, we contribute by providing information to managers, so that they can make decisions with more elements of assessment.

## Limitations and Future Research Lines

It can be observed that both methodologies significantly argue certain variables in the prediction of the future intentions of the customers of a sports center, but some limitations have also been found in this research. One limitation is the use of a non-probabilistic sample, as not all the population is represented universally. Even so, observing the sample quantity obtained, we estimate that the results obtained represent an approach to the object of study. In this sense, the way that the participants use the service has not been considered, so we cannot know if these emotional factors can be linked to the type of activities they engage in. On the other hand, the data obtained correspond to a specific type of service, without making a comparison with other types of centers, with other characteristics and peculiarities and belonging to different contexts. Therefore, in future research, we propose to extend the sample size, as well as to carry out probabilistic sampling. Similar contexts should also be analyzed, but within the national, even international, scope to observe differences between them, as well as to try to analyze public sports centers and to observe the main differences between private centers and to observe how their customers behave.

## Data Availability Statement

The raw data supporting the conclusions of this article will be made available by the authors, without undue reservation.

## Ethics Statement

Ethical review and approval was not required for the study on human participants in accordance with the local legislation and institutional requirements. Written informed consent to participate in this study was provided by the participants.

## Author Contributions

FG-P, IV, and FC-M contributed to the conception and design of the study. FG-P and MA organized the database. VP-G and FC-M performed the statistical analysis. FG-P wrote the first draft of the manuscript. VP-G, MA, FC-M, and IV wrote sections of the manuscript. All authors contributed to manuscript revision, read and approved the submitted version.

## Conflict of Interest

The authors declare that the research was conducted in the absence of any commercial or financial relationships that could be construed as a potential conflict of interest.
